# Neuroprotective Effect of *Yucca schidigera* Roezl ex Ortgies Bark Phenolic Fractions, Yuccaol B and Gloriosaol A on Scopolamine-Induced Memory Deficits in Zebrafish

**DOI:** 10.3390/molecules27123692

**Published:** 2022-06-08

**Authors:** Łukasz Pecio, Solomiia Kozachok, Ion Brinza, Razvan Stefan Boiangiu, Lucian Hritcu, Cornelia Mircea, Ana Flavia Burlec, Oana Cioanca, Monica Hancianu, Olga Wronikowska-Denysiuk, Krystyna Skalicka-Woźniak, Wiesław Oleszek

**Affiliations:** 1 Department of Biochemistry and Crop Quality, Institute of Soil Science and Plant Cultivation—State Research Institute, Czartoryskich 8, 24-100 Puławy, Poland; lpecio@iung.pulawy.pl (Ł.P.); wieslaw.oleszek@iung.pulawy.pl (W.O.); 2 Department of Natural Products Chemistry, Medical University of Lublin, 20-093 Lublin, Poland; kskalicka@pharmacognosy.org; 3 Department of Biology, Faculty of Biology, Alexandru Ioan Cuza University of Iasi, 700506 Iasi, Romania; ion.brinza@student.uaic.ro (I.B.); razvan.boiangiu@student.uaic.ro (R.S.B.); 4 Department of Pharmaceutical Biochemistry and Clinical Laboratory, Faculty of Pharmacy, “Grigore T. Popa” University of Medicine and Pharmacy, 16 University Street, 700115 Iasi, Romania; corneliamircea@yahoo.com; 5 Department of Drug Analysis, Faculty of Pharmacy, “Grigore T. Popa” University of Medicine and Pharmacy, 16 University Street, 700115 Iasi, Romania; ana-flavia.l.burlec@umfiasi.ro; 6 Department of Pharmacognosy, Faculty of Pharmacy, “Grigore T. Popa” University of Medicine and Pharmacy, 16 University Street, 700115 Iasi, Romania; oana.cioanca@gmail.com (O.C.); mhancianu@yahoo.com (M.H.); 7 Independent Laboratory of Behavioral Studies, Medical University of Lublin, 20-059 Lublin, Poland; olga.wronikowska-denysiuk@umlub.pl

**Keywords:** *Yucca schidigera*, Asparagaceae, spiro-flavostilbenoids, stilbenoids, polyphenols, zebrafish, anxiety, memory, cholinergic function, oxidative stress

## Abstract

*Y. schidigera* contains a number of unusual polyphenols, derivatives of resveratrol and naringenin, called spiro-flavostilbenoids, which have potent in vitro anti-inflammatory, antioxidant, and moderate cholinesterase inhibitory activities. To date, these compounds have not been tested in vivo for the treatment of neurodegenerative diseases. The aim of the present study was to evaluate the effects of both single spiro-flavostilbenoids (yuccaol B and gloriosaol A) and phenolic fractions derived from *Y. schidigera* bark on scopolamine-induced anxiety and memory process deterioration using a *Danio rerio* model. Detailed phytochemical analysis of the studied fractions was carried out using different chromatographic techniques and Nuclear Magnetic Resonance (NMR). The novel tank diving test was used as a method to measure zebrafish anxiety, whereas spatial working memory function was assessed in Y-maze. In addition, acetylcholinesterase/butyrylcholinesterase (AChE/BChE) and 15-lipooxygenase (15-LOX) inhibition tests were performed in vitro. All pure compounds and fractions under study exerted anxiolytic and procognitive action. Moreover, strong anti-oxidant capacity was observed, whereas weak inhibition towards cholinesterases was found. Thus, we may conclude that the observed behavioral effects are complex and result rather from inhibition of oxidative stress processes and influence on cholinergic muscarinic receptors (both 15-LOX and scopolamine assays) than effects on cholinesterases. *Y. schidigera* is a source of substances with desirable properties in the prevention and treatment of neurodegenerative diseases.

## 1. Introduction

*Yucca schidigera* Roezl ex Ortgies (Mojave/Mohave yucca) of the Asparagaceae family is native to the southwestern United States, Mexico and grows in hot and arid climates. The fruits and seeds of *Y. schidigera* (YS) have been historically documented and culturally transmitted as a food source for over 3000 years in the Mojave Desert [1]. Mojave yucca extract has been used by American Indians to soothe joint pain, bleeding, urethritis, and prostate inflammation; in addition, an infusion of the leaves and roots is recommended to treat asthma and headaches [2]. YS products have been approved by the U.S. Food and Drug Administration (FDA) and have Generally Regarded as Safe (GRAS) status [3,4]. Currently, YS products are used as flavorings and foaming agents in soft drinks and foods, as surfactants and preservatives in cosmetics, as nutraceuticals, as biopesticides and fertilizers for crops, and as animal feed additives [5]. YS extracts are commercially produced from pressed and dejuiced trunk and flowering stems or as dried powder from unprocessed yucca. The activity of the aforementioned commercial products is related to the presence of yucca steroidal saponins (furostanol- and spirostanol-types), accounting for about 10% of the dry weight [2,6].

YS bark is a byproduct that contains unique phenolic metabolites i.e., spiro-flavostilbenoids (biosynthesized from *2R*-naringenin and *trans*-resveratrol (RV) or *trans*-3,3′,5,5′-tetrahydroxy-4′-methoxystilbene (THMS)), which were previously only detected in the *Yucca* L. genus [7]. The spiro-compounds of YS include spiro-flavostilbenoids Yuccaols (Yus A-E), Yuccalide A (YueA), Gloriosaols (Glos A and C-E), and spiro-biflavonoids, namely Yuccalechins A-C [8]. Individual spiro-flavostilbenoids, RV, THMS, and the YS phenolic fraction were tested in vitro for their anti-inflammatory properties. RV had the strongest COX-2 inhibitory activity, followed by the phenolic fractions, YuB, YuA and YuC. YS fractions and RV moderately inhibited 5-lipoxygenase (5-LOX) pathway against leukotriene B4 formation [6]. The anti-inflammatory activity of YuC was also confirmed by Marzocco et al. [9] and Nakashima et al. [10] in macrophage cell line previously incubated with the compound and stimulated with lipopolysaccharide (LPS) after 1 h. Thus, YuC significantly inhibited nitric oxide (NO) release and inducible nitric oxide synthase (iNOS) protein expression through the transcription factor NF-κB (nuclear factor kappa-light-chain-enhancer of activated B cells). Moreover, YuE also effectively decreased the mRNA level of iNOS produced by LPS-induced macrophages. In the same assay, YuC and YuE significantly reduced the formation of interleukin IL-6 and IL-1β, at a concentration of 100 μM [10]. In another study, RV, THMS, YuA-C (tested at 25 μM), exerted anti-inflammatory effects in vitro in Kaposi’s sarcoma (KS) cells induced by vascular endothelial growth factor (VEGF). Yuccaols and simple stilbenes were able to significantly inhibit platelet-activating factor (PAF) biosynthesis and mitogen-induced proliferation [11]. The antiplatelet effects of the phenolic fraction of YS and its individual metabolites were also investigated in vitro. Thrombin-activated porcine platelet adhesion to collagen and fibrinogen, lysosome, protein and adenine nucleotide release were determined [12]. The most profound reduction in platelet adhesion and secretion in a dose-depended manner was attributed to RV and YS phenolic fraction.

The anti-inflammatory and anti-allergic effects of gloriosaols mixture were demonstrated in vivo in BALB/c mice with ovalbumin-induced airway hyperresponsiveness. The Glos mixture (30 mg/kg; p.o.) effectively reduced the levels of proinflammatory mediators, i.e., TNF-α (tumor necrosis factor-α), IL-6 and IL-13 in bronchoalveolar lavage fluid (BALF), which was comparable to the reference compound dexamethasone (30 mg/kg; p.o.) [13]. The antioxidant properties of the tested mixture and their effects on myeloperoxidase and malonaldehyde concentration in lung tissues and nitrite and nitrate levels in BALF were also investigated. These parameters were significantly decreased in mice supplemented with Glos.

The in vitro antioxidant effects of YuA-YuE, RV, THMS, and phenolic fraction from YS bark were investigated using β-carotene and linoleic acid autoxidation assays. As a result, the fraction together with individual spiro-flavostilbenoids and RV showed significant activity, higher than the standard compound BHT (2,6-di-*tert*-butyl-4-methoxyphenol) at 120 min of assay. THMS was inactive in this model. In contrast, THMS and its spiro-derivatives, Glos A-E, exhibited strong radical scavenging activity in the ABTS^•+^ assay. The tested gloriosaols had significantly stronger activity than the reference compound (quercetin), Yus A-E and RV [14,15].

**Figure 1:**
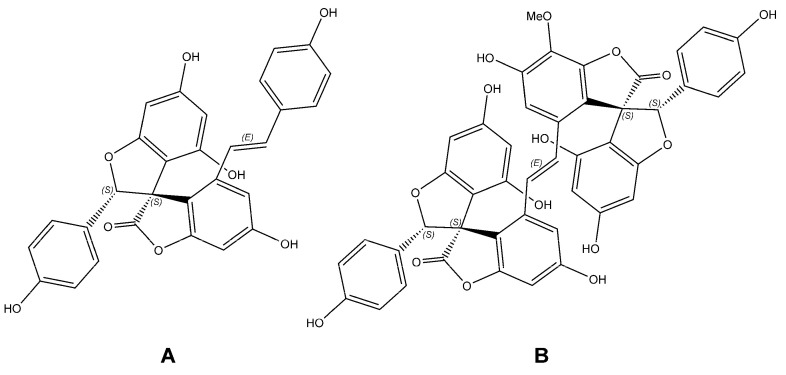
The structure of spiro-flavostilbenoids: (**A**) Yuccaol B (YuB); (**B**) Gloriosaol A (GloA).

In fact, the biological activity of spiro-flavostilbenoids is still undiscovered and requires in-depth studies, which is supported by the research results so far. On the other hand, their biosynthetic precursors, namely RV and naringenin, have been well studied in in vitro, ex vivo, in vivo and clinical trials for the prevention and treatment of neurodegenerative diseases, including Alzheimer’s disease (AD) and Parkinson’s disease (PD) [16]. Importantly for the treatment of these disorders, our recent study showed weak and moderate inhibitory activity of Yus and Glos against acetylcholinesterase (AChE) and butyrylocholinesterase (BChE) in in vitro assays, respectively, [7] and YuB and GloA (Figure 1) showed the highest inhibitory capacity against both enzymes. Their IC_50_ values against BChE were almost 2-fold (GloA) and 1.5 (YuB) lower than those of galantamine [7].

Our current study was designed to evaluate the neuropharmacological effects of spiro-flavostilbenoids and phenolic fractions derived from YS bark on scopolamine (Sco)-induced models of anxiety and amnesia in zebrafish (*Danio rerio*). In vivo bioassays were associated with in-vitro AChE/BChE and 15-LOX inhibition assays. This study is the first to evaluate the neuroprotective potential of YS phenolics. Either single spiro-flavostilbenoids—YuB and GloA—or phenolic fractions i.e., unpurified (YS unpur), purified (YS pur) and polymeric fractions (YS poly) were tested. Detailed phytochemical analysis of the studied fractions was carried out using different chromatographic techniques and NMR studies.

## 2. Results

### 2.1. Chemical Characterization of Y. schidigera Fractions. Quantitative Analyses

Phenolic fractions of YS i.e., YS unpur, YS pur, YS poly and single metabolites YuB and GloA were isolated from powdered yucca bark. The YS pur and YS poly were obtained from the unpurified fraction using the combination of gel filtration and silica gel chromatography. As a result, 10 g of YS unpur yielded 5.95 g of YS pur and 3.03 g of YS poly. In YS bark, the percentages of YS unpur, YS pur and YS poly fractions were 5.15%, 3.09% and 1.55%, respectively.

#### 2.1.1. Chemical Characterization of YS Unpur and YS Pur

Chemical characterization of YS unpur and YS pur phenolic fractions was performed using UHPLC-UV-MS analysis. Phenolic metabolites were identified from retention times and spectroscopic data (UV-Vis and mass spectrometry) of previously isolated YS standards [7,8]. The quantification of individual phenols was carried out by the external standard method, i.e., using *trans*-resveratrol and detection at 320 nm. As a result, 15 compounds were identified and quantified (Table 1). The sum of low-molecular weight phenolic compounds in YS unpur and YS pur was 32.4% and 50.3% of the dry residue, respectively. The major components of these fractions were THMS (48.85 ± 0.64 mg/g in YS unpur/73.64 ± 1.14 mg/g in YS pur), YuC (66.34 ± 1.00/103.96 ± 1.46 mg/g) and YuD (58.12 ± 0.83/90.42 ± 1.35 mg/g). Monomeric stilbenoids (THMS and RV) accounted for 17.6% of YS unpur identified phenolics and 17.1% of YS pur, flavonoids (dihydrokaempferol, naringenin, and kaempferol) accounted for 8.4% of YS unpur and 8% of YS pur. Spiro-flavostilbenoids in total accounted for nearly ¾ of all identified metabolites by content and the ratio of yuccaols (RV/THMS and naringenin dimers) to trimeric gloriosaols was 3.7:1.0 (Table 1).

#### 2.1.2. Chemical Characterization of YS Poly

Understanding the chemical composition of the YS poly fraction, which represents ⅓ of the YS unpur, was problematic due to, among other things, the lack of chromatographic peaks during LC-UV-MS analyses and after thiolysis [17], typically used to study the chemical composition of proanthocyanidins by depolymerization (data not shown). The lack of released flavan-3-ol monomers during thiolysis led us to think that YS poly is rather a polymer of THMS and naringenin, like the spiro-flavostilbenoids that predominate the YS unpur and YS pur fractions.

**Figure 2:**
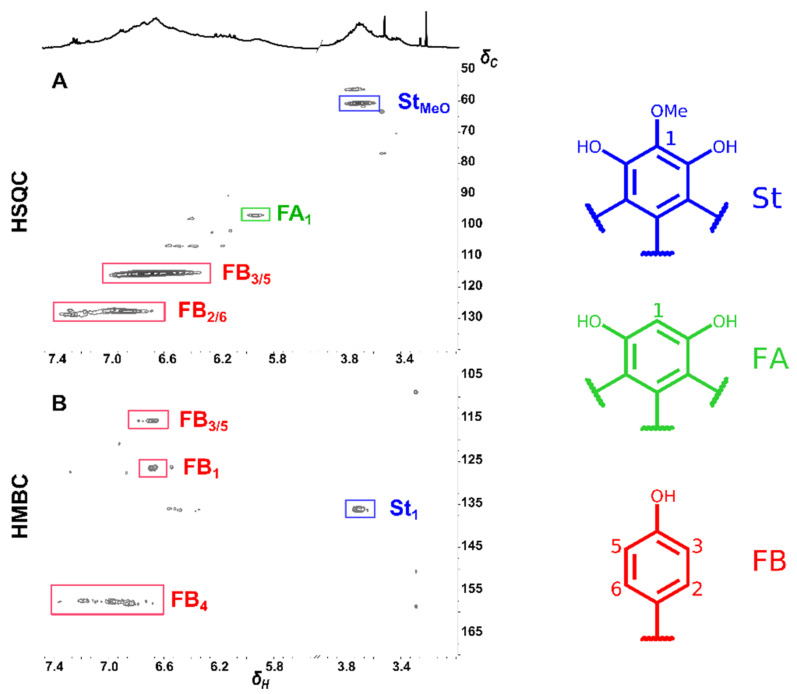
2D-HSQC- (**A**) and 2D-HMBC-NMR (**B**) spectra (in acetone-*d_6_*:D_2_O, 7:3) of the polymeric fraction (YS poly) isolated from *Y. schidigera* cortex. Top black trace: ^1^H NMR spectrum of YS poly.

Identification of such a complex mixture of compounds is beyond the scope of this publication, nevertheless we decided to perform 2D ^1^H-^13^C HSQC and ^1^H-^13^C HMBC NMR analyses, which gave the information regarding different stilbene (St) and flavanone (FA and FB) units present in the polymeric fraction of YS (Figure 2). Signals from methoxy (MeO) belonging to THMS stilbenic moiety at δ_C_/δ_H_ 60.66/3.56–3.86 and parahydroxyphenyl groups from ring B of flavanone moiety (FB) from C_2,6_/H_2,6_ at δ_C_/δ_H_ 127.6–128.9/6.65–7.36 and C_3,5_/H_3,5_ at δ_C_/δ_H_ 115.6/6.30–7.00 are dominant in the HSQC spectra, accompanied by cross peak from flavanone ring A (FA) at δ_C_/δ_H_ 96.9/5.87–6.00 (Figure 2A). The HMBC spectrum, on the other hand, shows the connection between the methoxy group and the signal of C_1_ (St_1_) at δ_C_ 136.1, and from protons FB_2/6_ to carbon FB_4_ at δ_C_ 157.3–158.0 (Figure 2B).

### 2.2. In Vitro Bioassays

#### 2.2.1. AChE/BChE Inhibition Assay

YS phenolic fractions were tested in vitro for their ability to inhibit cholinesterases. The ability to inhibit AChE/BChE was found to be low for the fractions YS unpur (EC_50_ = 188.76 ± 0.59/176.14 ± 1.14 µg/mL) and YS pur (EC_50_ = 419.45 ± 0.86/165.93 ± 0.44 µg/mL). Their effective concentrations are 7.7/7.6 (YS unpur) and 17/7.2 (YS pur) times higher than the corresponding reference compound galantamine (EC_50_ = 24.69 ± 0.09/23.07 ± 0.13 µg/mL) against AChE and BChE, respectively. In contrast, YS poly fraction was a moderate inhibitor of AChE (EC_50_ = 82.51 ± 0.20 µg/mL) and BChE (104.46 ± 0.59 µg/mL) (Tables S1 and S2, Supplementary Materials).

#### 2.2.2. 15-LOX Inhibition

YS fractions and pure compounds showed a promising ability to inhibit 15-LOX (Table S3), which was higher compared to that of the reference compound, vitamin C (EC_50_ = 21.52 ± 0.95 µg/mL). YS pur (EC_50_ = 8.76 ± 0.21 µg/mL) was the most effective inhibitor, with a value almost 2.5 times lower compared to ascorbic acid and very similar to that recorded for YuB (EC_50_ = 9.66 ± 0.12 µg/mL), then GloA (EC_50_ = 12.34 ± 0.48 µg/mL) and YS poly (12.78 ± 1.74 µg/mL), while the least was noticed for YS unpur (EC_50_ = 17.65 ± 0.13 µg/mL).

### 2.3. In Vivo Bioassays

In vivo studies evaluated the memory and anxiety-like behaviors exerted by YS pur and YS poly, and pure YuB and GloA in a model of Sco-induced cognitive deficits and increase in anxiety level in zebrafish (*Danio rerio*). Y-maze (memory evaluation) and NTT (anxiety assessment) tests were performed.

#### 2.3.1. Y-Maze Test

The locomotor tracking pattern in the control group was demonstrated by normal swimming all over the Y-maze tank (Figure S1). The Sco group exhibited deficits in the response to novelty, which is represented by abnormal tracking pattern, as assessed by less exploration in the novel arm. The locomotor tracking pattern of YS pur- and YS poly-, YuB- and GloA-treated groups at the doses (1, 3 and 5 μg/L) showed attenuation of Sco effect and a swimming pattern nearly similar to control group as shown in Figure S1, Supplementary Materials.

**Figure 3:**
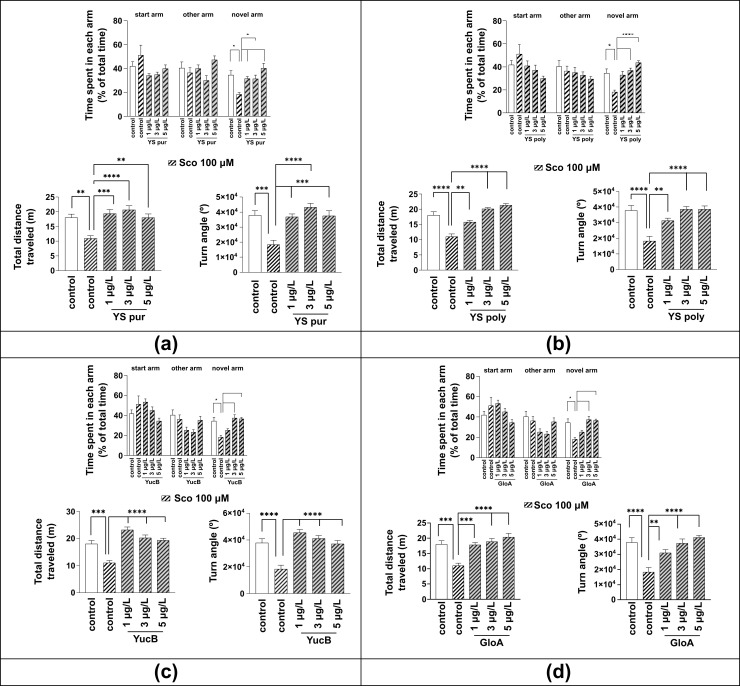
The effects of YS preparations (1, 3 and 5 μg/L) on memory and locomotor activity in scopolamine (Sco)-treated zebrafish in the Y-maze test. Memory: time spent in each arm (start, other and novel arm); Locomotion: total distance traveled and Turn angle of zebrafish in the tank in the control—untreated group; group treated with scopolamine (Sco 100 µM); groups treated with Sco (100 µM) and (**a**) YS pur—purified fraction; (**b**) YS poly—polymeric fraction; (**c**) YuB—yuccaol B; and (**d**) GloA—gloriosaol A. Values are means ± S.E.M. (*n* = 10). For Tukey’s post hoc analyses * *p* < 0.05, ** *p* < 0.01, *** *p* < 0.001, **** *p* < 0.0001.

Figure 3a shows that YS pur affected memory and locomotor activity in Sco-treated zebrafish in the Y-maze test. Two-way ANOVA showed significant effect of the treatment (F (4, 135) = 4.775; *p* = 0.0012), time spent on arms (F (2, 135) = 7.644; *p* = 0.0007 and interactions (F (8, 135) = 4.345; *p* = 0.0001). Tukey’s post hoc test confirmed that Sco decreased the time spent in the novel arm (*p* < 0.05) in comparison with control group. As Sco-exposed zebrafish explored the novel arm less, this suggests deficits in response to novelty and impaired memory performance. YS pur significantly improved memory deficits as evidenced by increased time spent in the novel arm (1 and 5 μg/L—*p* < 0.01 and 3 μg/L—*p* < 0.05) compared with the Sco-treated group. Moreover, a statistically significant effect of treatment was observed in total distance traveled (one-way ANOVA F (4, 45) = 11.21, *p* < 0.0001) and turn angle (one-way ANOVA F (4, 45) = 9.46, *p* < 0.0001). Hypolocomotion induced by Sco treatment was observed as a decrease in distance travelled (*p* < 0.01) and turn angle (*p* < 0.001). YS pur prevented Sco-induced hypolocomotion by significantly increasing the total distance traveled in the tank (*p* < 0.001 for 1 μg/L, *p* < 0.0001 for 3 μg/L and *p* < 0.01 for 5 μg/L) and turn angle (*p* < 0.001 for 1 μg/L, *p* < 0.0001 for 3 μg/L and *p* < 0.001 for 5 μg/L) compared to the Sco-treated group.

Figure 3b shows that YS poly affected memory and locomotor activity in Sco-treated zebrafish in the Y-maze test. Two-way ANOVA showed significant effect of the time spent in arms (F (2, 162) = 9.118, *p* = 0.0002) and interaction (F (10, 162) = 4.833; *p* < 0.0001) without treatment (F (5, 162) = 0.5385; *p* = 0.7469). Tukey’s post hoc test confirmed that Sco decreased the time spent in the novel arm (*p* < 0.05) in comparison with control group. YS poly significantly increased the time spent in the novel arm in a dose-dependent manner (3 μg/L—*p* < 0.01 and 5 μg/L—*p* < 0.0001) compared to Sco-treated group. Moreover, the statistically significant effect of treatment was observed in total distance traveled (one-way ANOVA F (4, 45) = 27.14, *p* < 0.0001) and the turn angle (one-way ANOVAF (4, 45) = 13.42, *p* < 0.0001). Sco induced decrease in the distance travelled and turn angle (*p* < 0.0001). YS poly prevented Sco-induced hypolocomotion by significantly increasing total distance traveled in the tank and turn angle (*p* < 0.01 for 1 μg/L, *p* < 0.0001 for 3 μg/L and 5 μg/L) as compared with Sco-treated group.

Figure 3c shows that YuB affected memory and locomotor activity in Sco-treated zebrafish in the Y-maze test. Two-way ANOVA showed a significant effect of the time spent in arms (F (2, 162) = 31.45; *p* < 0.0001)) and interaction (F (10, 162) = 5.287; *p* < 0.0001) without treatment (F (5, 162) = 0.5616; *p* = 0.7293). Tukey’s post hoc test confirmed that Sco decreased the time spent in the novel arm (*p* < 0.05) in comparison with control group. YuB significantly increased the time spent in the novel arm (3 μg/L—*p* < 0.01 and 5 μg/L—*p* < 0.0001) as compared with Sco-treated group. Moreover, the statistically significant effect of treatment was observed in total distance traveled (one-way ANOVA F (4, 45) = 20.17, *p* < 0.0001) and the turn angle (one-way ANOVA F (4, 45) = 15.86, *p* < 0.0001). Sco treatment-induced hypolocomotion was observed as decreased distance travelled (*p* < 0.001) and turn angle (*p* < 0.0001). YuB prevented Sco-induced hypolocomotion, significantly increasing the total distance traveled in the tank and turn angle at all concentrations (*p* < 0.0001) as compared with Sco-treated group.

Figure 3d shows that GloA affected memory and locomotor activity in the Sco-treated group in the Y-maze test. Two-way ANOVA showed a significant effect of the time spent in arms (F (2, 162) = 31.45; *p* < 0.0001) and interactions (F (10, 162) = 5.287; *p* < 0.0001) without treatment effect (F (5, 162) = 0.5616; *p* = 0.7293). Tukey’s post hoc test confirmed that Sco decreased the time spent in the novel arm (*p* < 0.05) in comparison with control group. GloA significantly improved memory deficits induced by Sco observed as an increase in the time spent in the novel arm (3 μg/L—*p* < 0.01 and 5 μg/L—*p* < 0.0001) as compared with Sco-treated group. Moreover, the statistically significant effect of treatment was observed in total distance traveled (one-way ANOVA F (4, 45) = 12.25, *p* < 0.0001) and the turn angle (one-way ANOVA F (4, 45) = 12.44, *p* < 0.0001). Sco treatment-induced hypolocomotion was observed as decreased distance travelled (*p* < 0.001) and turn angle (*p* < 0.0001). GloA prevented Sco-induced hypolocomotion by significantly increasing the total distance traveled in the tank (*p* < 0.001 for 1 μg/L, *p* < 0.0001 for 3 μg/L and 5 μg/L) and turn angle (*p* < 0.01 for 1 μg/L, *p* < 0.0001 for 3 μg/L and 5 μg/L) as compared with Sco-treated group.

All YS preparations tested exhibited memory-enhancing properties in the Y-maze task. The improvement in cognitive function was accompanied by an increase in locomotor activity.

#### 2.3.2. Novel Tank Diving Test (NTT)

**Figure 4:**
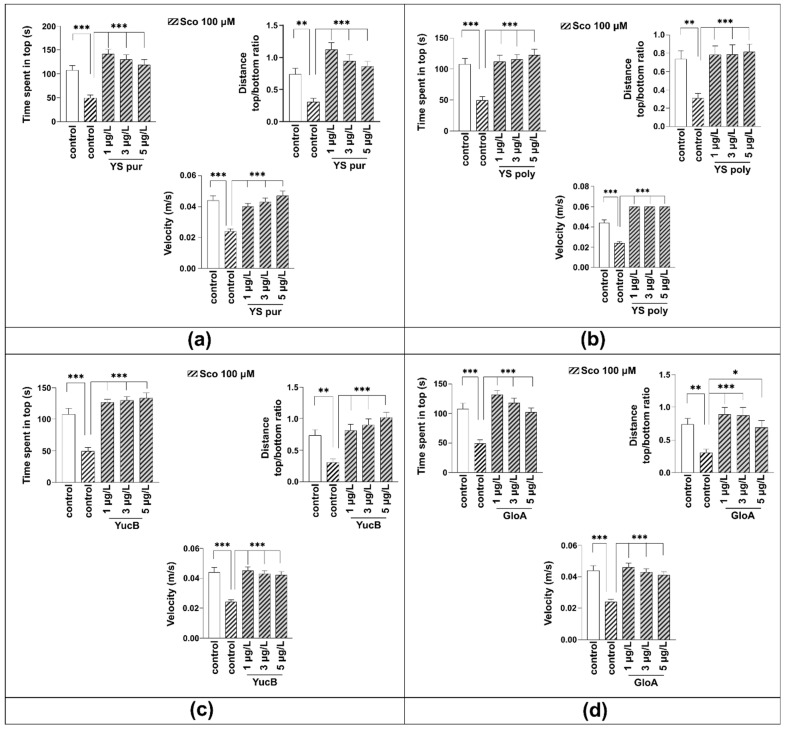
The influence of *Y. schidigera* preparations in scopolamine (Sco)-treated zebrafish on locomotor activity and anxiety level observed in the Novel Tank Diving Test (NTT). Anxiety response: time spent in the top (s); Locomotion: distance top/bottom ratio; and Velocity (m/s) in the control group; group treated with Sco 100 µM; groups treated with Sco (100 µM) and yucca preparations at 1, 3, 5 µg/L: (**a**) YS pur—purified fraction; (**b**) YS poly—polymeric fraction; (**c**) YuB—Yuccaol B; (**d**) GloA—Gloriosaol A. Values are means ± S.E.M. (*n* = 10). For Tukey’s post hoc analyses * *p* < 0.05, ** *p* < 0.01 and *** *p* < 0.001.

In the NTT test, Sco treatment significantly decreased the time spent in the top zone of the tank (*p* < 0.0001) as compared with the control group, suggesting Sco-induced anxiogenic effect (Figure 4). Sco treatment induced a hypolocomotor effect as compared with the control group observed as a decrease in the distance top/bottom ratio (*p* < 0.01) and the velocity (*p* < 0.001).

One-way ANOVA revealed a significant effect of: the YS pur (1, 3 and 5 μg/L, Figure 4a) treatment on the time spent in the top zone of the tank (F (4, 45) = 46.52, *p* < 0.0001), on distance top/bottom ratio (F (4, 45) = 61.88, *p* < 0.0001), and on velocity (F (4, 45) = 12.22, *p* < 0.0001); YS poly (1, 3 and 5 μg/L, (Figure 4b) treatment on the time spent in the top zone of the tank (F (4, 45) = 11.23, *p* < 0.0001), on distance top/bottom ratio (F (4, 45) = 5.714, *p* = 0.0008), and on velocity (F (4, 45) = 105.3, *p* < 0.0001); YuB (1, 3 and 5 μg/L, Figure 4c) treatment on the time spent in the top zone of the tank (F (4, 45) = 24.57, *p* < 0.0001), on distance top/bottom ratio (F (4, 45) = 10.21, *p* < 0.001), and on velocity (F (4, 45) = 12.88, *p* < 0.0001); and GloA (1, 3 and 5 μg/L, Figure 4d) treatment one-way ANOVA revealed a significant effect of the treatment on the time spent in the top zone of the tank (F (4, 45) = 17.55, *p* < 0.0001), on distance top/bottom ratio (F (4, 45) = 5.797, *p* = 0.0008), and on velocity (F (4, 45) = 13.62, *p* < 0.0001).

In YS pur and YS poly and pure compounds the anxiolytic-like effect was noticed by increasing the time spent in the top zone of the tank (*p* < 0.001) as compared with the Sco-alone treated animals. Moreover, treatment with the tested fractions and compounds (1, 3 and 5 μg/L) prevents the hypolocomotor effect of Sco on the velocity (*p* < 0.001) as compared with Sco-alone-treated fish. An increase in the distance top/bottom ratio was also observed for YS pur, YS poly, and YuB (*p* < 0.001) and GloA (*p* < 0.001 for 1 and 3 μg/L, *p* < 0.05 for 5 μg/L).

Overall, YS pur and YS poly as well as YuB and GloA showed anxiolytic effects. The tested YS fractions/compounds were safe for zebrafish at the selected doses.

## 3. Discussion

Numerous studies show the implication of oxidative stress in the physiopathology of neurodegenerative diseases such as AD and PD. The imbalance between pro-oxidant compounds and antioxidant endogenous systems can be re-established by, for example, administering plant extracts rich in polyphenol carboxylic acids, flavonoids and anthocyanins [18]. In our experiments, we focused on 15-LOX, an enzyme which catalyzes the oxidation of unsaturated fatty acids, leading to the formation of peroxides involved in oxidative stress, inflammation and neurodegeneration [19,20]. YS unpur, YS pur, YS poly, YuB and GloA showed a strong in vitro 15-LOX inhibition capacity, stronger compared to vitamin C. The results obtained are consistent with previous studies [13,14,15]. This can be explained by the presence of compounds with hydrophobic functional groups, which are able to reach the active site of the enzyme, as well as the presence of compounds with hydrophilic groups, which donate protons and electrons and block the redox reaction. The active phenolic metabolites of YS can block the enzyme through various mechanisms: (1) by blocking the reversible redox conversion Fe^2+^ → Fe^3+^—compounds that donate hydrogen act as inhibitors in this mechanism; (2) by blocking access of the substrate (linoleic acid) to the enzyme active site—this applies to compounds with significant and large lipophilic groups; (3) by modifying the spatial structure of the enzyme, which indirectly leads to the modification of the enzyme’s active site and subsequently to the lack of interaction between enzyme and substrate—attributed to compounds with large lipophilic groups, compounds with positively or negatively ionized functional groups, and compounds capable of forming hydrogen bonds with functional groups present in the enzyme structure [19].

Yucca phenolic fractions showed weak in vitro inhibition against AChE and moderate inhibition against BChE. These results correspond with our previous studies [7] on the inhibitory capacity of pure YS spiro-flavostilbenoids, stilbenoids and flavonoids. In brief, flavonoids (naringenin, dihydrokaempferol) and one of the major stilbenoids, THMS, did not inhibit cholinesterase activity (Table S4). The major spiro-flavostilbenoids, namely: YuC and YuD showed very low activity, a galantamine-like effect was obtained at 74/1.9 times higher doses (for AChE/BChE) of YuC, and 193/5.5 of YuD. YuB and GloA were the most active isolates, their effective concentrations compared to galantamine were 19 and 20 times higher for AChE, respectively. In contrast, YuB and GloA were more active than galantamine in assays with BChE. Cholinesterase inhibitors have significant beneficial effects on attention, information acquisition, memory and language, but do not improve visuospatial abilities. Intense inhibition of these enzymes may worsen symptomatology in patients with cognitive deficits and PD [21].

Since weak cholinesterase inhibition was observed, we decided to search for mechanisms other than cholinergic ones underlying the behavioral effects of the tested fractions and compounds. Accordingly, we were first to investigate the effects of yucca spiro-flavostilbenoids on Sco-induced memory deterioration and anxiogenic effects in adult zebrafish. Sco, by blocking cholinergic muscarinic receptors, affects the cholinergic system. The negative effects of Sco (100 µM) i.e., decreased locomotor activity, deficits in response to novelty, increased anxiety, were attenuated in zebrafish that were incubated with YS pur, YS poly, YuB and GloA (at concentrations of 1, 3, 5 µg/L). Yucca preparations were active in both Y-maze and NTT-tests. No visible developmental defects were observed after incubation with the tested substances, suggesting that YS phenolic fractions and pure compounds are not toxic. This is consistent with the GRAS status of the YS products.

To our knowledge, there are no literature data on the neuroprotective properties of spiro-flavostilbenoids and yucca phenolics tested in vivo. YS spiro-flavostilbenoids are composed of THMS/RV and naringenin units, as well as polymers—THMS and naringenin. Based on scientific reports [22,23,24], we hypothesized that dimers, trimers, i.e., spiro-flavostilbenoids and their polymers are metabolized by the gut microbiota to monomeric units that reach the blood serum and brain [16,25], causing the observed biological effect of YS phenolics. Wang et al. described that the a grape-derived polyphenolic preparation was metabolized to corresponding monomeric forms of catechin and epicatechin conjugates (glucuronides and methylated ones), which exerted direct neuroprotective effects [22]. The monomeric derivatives were accumulated from blood into mouse brain tissue, and the bioavailability of methylated glucuronides was higher. On the other hand, the same authors pointed out the lack of activity of oligo- and polymeric fractions, due to the low bioavailability of catechin and epicatechin conjugates. This indicates different absorption mechanisms of spiro-flavostilbenoids compared to flavan-3-ols.

Our results indicate a positive effect of YS pur and YS poly fractions along with YuB and GloA on Sco-induced memory deterioration and anxiogenic effects. Both pure compounds (YuB and GloA) and fractions (YS pur and YS poly) contain single or mixtures of compounds with similar chemical structures (spiro-flavostilbenoids) which may have been converted into similar metabolites by intestinal metabolism of zebrafish microbiota, and then these metabolites were distributed to fish brains through the blood-brain barrier (BBB). This theory may be supported by the high activity of YS poly, which contains mostly medium-to-high molecular weight compounds and thus is most likely incapable of passively penetrating the BBB [26]. However, to know what compounds actually penetrated the BBB requires further studies to be performed. The obtained in-vitro and in-vivo results highlight the neuroprotective action of yucca phenolics and link it to antioxidant and anti-inflammatory activities, as well as to muscarinic receptor activation.

The mentioned neuroprotective mechanism is also reported for RV and its derivatives [16,18]. RV exhibits potent anti-oxidant activity (scavenges reactive oxygen species, increases glutathione levels, improves endogenous antioxidants), modulates neuroinflammation (attenuates inflammatory mediators NO, TNF-α, IL, MCP-1, reduces NF-kB transcription and microglia activation) and prevents neuronal death by activating SIRT1 [27], and activates cholinergic pathway [16]. Methoxylation of RV units (e.g., pterostilbene) enhances the metabolic stability and bioavailability due to increased lipophilicity, cellular uptake, and oral absorption compared to RV [16]. Pterostilbene improves cognitive deficits in the older rodents (19-month-old male Fischer 344 rats) in the Moris water maze test by increasing muscarinic receptor sensitivity and calcium buffering [28]. An in vivo study showed that dietary supplementation with pterostilbene or RV improved cognitive function in mice with age-related AD (SAMP8) [25]. Pterostilbene was more potent than RV in activating endogenous antioxidant (increased manganese superoxide dismutase (MnSOD) activity) and anti-inflammatory (reduced NFκβ p65 levels) mechanisms by upregulating peroxisome proliferator-activated receptor (PPAR) alpha activity. Moreover, tau phosphorylation was decreased by pterostilbene, not RV.

The neuroprotective effects of naringenin are well studied in in vitro and in vivo models, and its multi-target mechanism of action is well documented [29]. Zhang et al. [30] found that naringenin promotes microglia polarization towards the M2 anti-inflammatory phenotype (upregulation of mediators such as arginase-1, transforming growth factor-β (TGF-β), IL-4, and IL-10). On the other hand, this flavanone attenuates the release of proinflammatory substances (TNF-α, IL-1β, iNOS) and inhibits LPS-induced microglial activation. Naringenin showed potent anti-oxidant activity at a dose of 50 mg/kg (p.o.) in male Wistar rats co-administrated with 50 mg/kg iron-dextran injection over 4 weeks. Naringenin supplementation reduced iron-induced neurotoxicity and anxiety-like behavioral deficit in rodent [31]. The tested flavanone exhibited neuroprotective effects by attenuation of reactive oxygen species (ROS) formation, increasing endogenous antioxidant capacity (significantly preventing the alterations in the activities of superoxide dismutase (SOD), catalase (CAT) and glutathione peroxides (GPx)), upregulating AChE activity and protecting against oxidative DNA damage mediated by apoptosis in the cerebral cortex. However, naringenin has poor bioavailability and low cerebral accessibility [29].

## 4. Materials and Methods

### 4.1. Chemicals and Reagents

Methanol, ethyl acetate, *n*-hexane as well as *tert*-BuOH, all of analytical reagent grade, were purchased from Fisher Chemical (Loughborough, UK) and Merck (Darmstadt, Germany), respectively. Acetonitrile and methanol (LC-MS grade) were purchased from Merck (Darmstadt, Germany), while MS-grade formic acid and other chemicals were purchased from Sigma-Aldrich (Steinheim, Germany). Ultrapure water was prepared using a Milli-Q water purification system (Millipore, Milford, MA, USA). Yuccaols A-E, Yuccalide A, gloriosaols A, C-E, and THMS were in-house prepared at the Department of Biochemistry and Crop Quality, IUNG [8]. Trans-resveratrol (Sigma Aldrich, Steinheim, Germany, >99% HPLC based) was used as a group standard for determination of yucca phenolics.

NMR spectra were recorded at 25 °C in the mixture of acetone-*d_6_* (99.9% D) and deuterated water (D_2_O, 99.96% D)—^1^H, HSQC and HMBC spectra or DMSO-*d_6_* (99.8%D) for the determination of purity of isolated compounds and fractions, and were purchased from Merck (Darmstadt, Germany).

Reagents for bioassays were di- and monosodium phosphates, 5,5′-dithiobis (2-nitrobenzoic acid) (DTNB), dimethyl sulfoxide (DMSO), acetylcholinesterase from Electrophorus electricus (electric eel), V-S type, butyrylcholinesterase from equine serum, acetylthiocholine iodide (ATCI), butyrylthiocholine iodide (BTCI); boric and linoleic acids, sodium hydroxide, *Glycine max* (soybean) 15-lipoxygenase (EC 1.13.11.12, CAS Number: 9029-60-1), ascorbic acid were bought from Sigma-Aldrich (Steinheim, Germany). Scopolamine and galantamine were obtained from ThermoFisher Scientific (Darmstadt, Germany).

### 4.2. Instruments

Gamma 2–16 LSC freeze dryer, Martin Christ Gefriertrocknungsanlagen GmbH, Germany. Waters ACQUITY UPLC chromatograph (Waters Corp., Milford, MA, USA) equipped with a binary pump and MS and DAD detectors. Prep- (Laborota 20 control automatic) and medium-scale (Hei-VAP) rotary evaporators (Heidolph, Germany). Nuclear magnetic resonance (NMR) spectrometer—an Avance III HD Ascend 500 MHz (Bruker BioSpin, Rheinstetten, Germany). The pH-meter (Hanna Instruments, Luton, UK), UV-Vis ABL&E Jasco V550 spectrophotometer (Cluj-Napoca, Romania), and Tetratec^®^ air pumps (Tetra, Melle, Germany) have been used in in vitro and in vivo bioassays.

### 4.3. Extraction and Isolation

The plant material—*Yucca schidigera* Roezl ex Ortgies bark was purchased from a commercial source (Desert King Int., Chula Vista, CA, USA). The extraction of unpurified phenolic fraction and isolation procedures of single stilbenoids were described in detail in our previous work [8]. Briefly, the powdered material was extracted 3 times with a new portion of 100% MeOH in 15-fold excess, using an ultrasonic bath (Polsonic 33, Warsaw, Poland) for 20 min of each series. All the procedures were performed at ambient temperature (20–21 °C) and in the dark. The partially evaporated extract was dissolved in water and defatted with *n*-hexane, then water-methanolic fraction was evaporated at 35 °C to eliminate the methanol. Subsequently the water fraction was extracted with ethyl acetate in the separatory funnel, evaporated and finally lyophilized. The obtained reddish-brown powder was YS unpur phenolic fraction, which was tested only in in vitro bioassays.

From this YS pur phenolic fraction was obtained using following procedure. A total of 1.24 g of YS unpur was dissolved in pure MeOH and loaded on a glass column (100 × 44 mm) filled with Sephadex LH-20 (40–120 µm, Sigma-Aldrich, Steinheim, Germany), eluted with 100% MeOH (500 mL, Fr4-12) and with 70% acetone solution (1000 mL, Fr13-20) at a flow rate of 25 mL min^−1^. Fraction Fr4-12 contained mostly stilbenoids and flavonoids, while Fr13-20—only polymeric compounds. Subsequently, Fr4-12 (1.14 g) was loaded on silica gel cartridge (25 µm, Biotage SNAP Ultra 50 g column 50 × 150 mm, Uppsala, Sweden) and eluted with step gradient of toluene:acetone solution (flow rate of 55 mL min^−1^), i.e., 50:50 (800 mL, Fr4-12_A) followed by 30:70 (600 mL, Fr4-12_B). Finally, the absorbed polymeric compounds were washed out with pure MeOH (400 mL, Fr4-12_C). Fr4-12_A contained stilbenoids and flavonoids—YS pur fraction, Fr4-12_B and Fr4-12_C were joined with Fr13-20 giving YS poly fraction. As a result, the beige-colored YS pur fraction (59.5% of YS unpur) and deep red-brown colored YS poly (30.3% of YS unpur) were obtained.

YuB and GloA were isolated from the YS unpur phenolic fraction as documented in [8]. YS pur, YS poly, YuB and GloA were tested either in vitro or in vivo.

### 4.4. NMR Spectroscopy, Purity of the Studied Fractions and Compounds

Multidimensional spectra (2D HSQC, 2D HMBC) were recorded with 12 mg of YS poly fraction dissolved in 0.65 mL of acetone-*d_6_*:D_2_O (7:3, *v*/*v*). Spectra were calibrated to the residual signal of acetone (δ_C_/δ_H_ 29.8/2.05). The HSQC experiment used Bruker’s standard hsqcedetgpsisp2.3 pulse program, the HMBC experiment used Bruker’s standard clhmbcetgpl3nd pulse program.

The samples were tested for their purity using NMR method (ERETIC2) by the application of external standard (3,5-dihydroxybenzoic acid) [32]. During the measurements, we learned that samples contained substantial and varying amounts (8–22% *w*/*w*) of *t*-BuOH—the solvent used for the freeze-drying process. YuB contained 15% *w*/*w* of *t*-BuOH, GloA 8%, YS unpur fraction 16%, YS pur fraction 22%, and YS poly fraction 9%.

### 4.5. Quantitative Analyses

The UHPLC-UV-MS was carried out on a Waters ACQUITY UPLC system (Waters Corp., Milford, MA, USA), comprising a binary pump system, sample manager, column manager, and PDA detector (Waters Corp., Milford, MA, USA). The acquisition and data processing were performed using Waters MassLynx software v.4.1. The samples were chromatographed on a BEH C18 column (100 mm × 2.1 mm i.d., 1.7 μm, Waters Corp., Milford, MA, USA), which was kept at 40 °C. The flow rate was adjusted to 400 µL min^−1^. The following solvent system: mobile phase A (0.1% formic acid in Milli-Q water, *v*/*v*) and mobile phase B (0.1% formic acid in MeCN, *v*/*v*) was applied. The gradient program was as follows: 0–2.0 min, 14% B; 2.0–21.0 min, 14–30% B (Waters convex gradient nr 6); 21.0–21.1 min, 30–99% B; 21.1–23.0 min, 99% B; 23.0–23.1 min, 99–14% B; 23.1–25.0 min, 14% B. Samples were thermostatted at 8 °C in the sample manager. The injection volume of the sample was 2.5 μL (partial loop with needle overfill mode). Strong needle wash solution (1:1:1, MeOH-MeCN-*i*PrOH, *v*/*v*/*v*) and weak needle wash solution (5:95, MeCN–H_2_O, *v*/*v*) were used. The detection wavelength was set at 320 nm (3.6 nm resolution) for the quantification of phenolics, at a 5 points s^−1^ rate. The MS analyses were carried out on a Waters TQ Detector, tandem quadrupole atmospheric pressure ionization (API) mass spectrometer equipped with a Z-spray electrospray interface (Waters Corp., Milford, MA, USA). The following instrumental parameters were used for ESI-MS analysis of all phenolic compounds (negative ionization mode): capillary voltage, 2.8 kV; cone voltage, 45 V; desolvation gas, N_2_ 900 L h^−1^; cone gas, N_2_ 100 L h^−1^; source temp. 150 °C, desolvation temp. 450 °C. Compounds were monitored in Selected Ion Monitoring mode (*m/z* for THMS—273.1; RV—227.1; YuC-YuE, YueA—541.1; YuA/YuB—495.1 and for Glos—809.2). Trans-resveratrol (Sigma Aldrich, >99% HPLC based) was used as a group standard for the determination of stilbenoids. Additionally, the molar absorption coefficients of RV (34,715 L/mol·cm^−1^), yuccaol B (29,220 L/mol·cm^−1^), gloriosaol A (25,474 L/mol·cm^−1^) naringenin (7000 L/mol·cm^−1^), dihydrokaempferol (10,000 L/mol·cm^−1^) and kaempferol (10,621 L/mol·cm^−1^) in 70% MeOH (*v*/*v*) were measured spectrophotometrically (*n* = 3, 3 levels at 0.01, 0.02 and 0.05 mM) to obtain high accuracy conversion factor for yuccaols (1.188) and gloriosaols (1.363), naringenin (4.959), dihydrokaempferol (3.472), and kaempferol (3.269).

The calibration curve was constructed with 9 points in the range of 0.0011–1.0953 mM. The working calibration curve was expressed with C_M_ = 1.240 × 10^−6^ × A − 3.758 × 10^−4^, where C_M_ is molar concentration in mM (10^−3^ mol/L) and A is area of the UV-chromatographic peak. The correlation coefficient for this curve was R^2^ = 0.99991 and the linearity range was 0.0022–0.5477 mM.

### 4.6. In Vitro Bioassays

#### 4.6.1. Cholinesterase (AChE/BChE) Inhibition Assay (Modified Ellman’s Method)

Briefly, 0.56 mL phosphate buffer 0.1M (pH 8) is mixed with 0.08 mL DTNB solution in phosphate buffer 0.1M (pH 8), 0.04 mL samples i.e., YS unpur, YS pur, YS poly solution (of different concentrations, in DMSO) and 0.08 mL enzyme solution (AChE or BChE) 2 UI/mL in phosphate buffer 0.02M (pH 7). The mixture is homogenized and maintained at 25 °C for 15 min, after which 0.04 mL ATCI or BTCI (considering the analyzed enzyme) is added and the absorbance of the solution is measured at 412 nm, for 5 min [33].

% inhibition = (A_EFI_ − A_ECI_) × 100/A_EFI_,

where: A_EFI_—is the difference between the absorbance of the enzyme solution without inhibitor after 5 min and the initial absorbance of the same solution (0 s); A_ECI_—represents the difference between the absorbance of the enzyme solution treated with inhibitor after 5 min and the initial absorbance of the same solution (0 s).

For each sample, the EC_50_ value was calculated and expressed as μg/mL final solution. Galantamine was used as a positive control.

#### 4.6.2. Lipoxygenase (15-LOX) Inhibition Activity (Modified Malterud Method)

Briefly, 0.9 mL borate buffer solution 0.1 M (pH 9) is mixed with 0.05 mL lipoxygenase solution in borate buffer pH 9 and with 0.05 mL samples i.e., YS unpur, YS pur, YS poly, YuB and GloA solution (of various concentrations, in DMSO). The mixture is left to stand for 10 min at room temperature, after which 2 mL linoleic acid solution 0.16 mM in borate buffer pH 9 is added. For each sample, a blank is prepared by substituting the enzyme solution with 0.05 mL borate buffer pH 9 solution. The absorbance of the solution was measured at 234 nm, in the 0–120 s interval [34].

% activity = (A_EFI_ − A_ECI_) × 100/A_EFI_,

where: A_EFI_—is the difference between the absorbance of the enzyme solution without inhibitor at 90 s and the absorbance of the same solution at 30 s; A_ECI_—is the difference between the absorbance of the enzyme solution treated with inhibitor at 90 s and the absorbance of the same solution at 30 s.

For each sample, the EC_50_ value was calculated and expressed as μg/mL final solution. Vitamin C was used as a positive control.

### 4.7. In Vivo Bioassays

#### 4.7.1. Animals

Adult zebrafish (*Danio rerio*) wild-type short-fin strain of both sexes (50:50, male:female ratio), 3–4-months old and 3–4 cm long, were purchased from an authorized commercial supplier (Pet Product S.R.L., Bucharest, Romania). The zebrafish were acclimatized in the experimental room for at least 14 days.

The fish were divided by 10 in a group and stored in 24 L thermostatted (26 ± 1 °C) tanks, kept under water filtration and aeration (7.20 mg O_2_/L) using Tetratec^®^ air pumps (Tetra, Melle, Germany). The animals were maintained on 14/10 h light/dark cycle and were fed twice a day with Norwin Norvitall flake (Norwin, Gadstrup, Denmark).

#### 4.7.2. Behavioral Assay

In behavioral studies, acclimatized zebrafish were randomly assigned to the control—untreated group; the Sco (100 µM) treated group; groups treated with Sco 100 µM and the studied samples i.e., YS pur, YS poly, YuB and GloA (at doses 1, 3, and 5 µg/L). The doses of the tested yucca preparations as well as Sco were selected according to our previous studies [35]. The tested doses (1, 3, and 5 µg/L) were diluted with 1% DMSO solution and administered to zebrafish by immersion for 1 h, once daily for 8 days, while Sco (100 µM) was administered for 30 min before each behavioral test. The control group was immersed only in unchlorinated water with a 1% DMSO solution. The animals were then transferred individually to a Y-maze glass tank or a trapezoid-shaped test tank filled with home tank water. Their swimming behavior was recorded with a Logitech C922 Pro HD Stream webcam (Logitech, Lausanne, Switzerland), and the recordings were analyzed using ANY-maze software v6.3 (Stoelting Co., Wood Dale, IL, USA).

All experiments were carried out following scrutiny by the Ethics Committee on Animal Research of the Alexandru Ioan Cuza University of Iasi, Faculty of Biology (Iasi, Romania) under license no. 02/30.06.2020 and fully complied with the Directive 2010/63/EU of the European Parliament and of the Council of 22 September 2010 on the safety of animals. The health status and the well-being of all animals involved in the research have been tested regularly during the behavioral tests. No procedures have caused serious pain or long-lasting damage to the zebrafish, and no experimental subject has died during the experimental procedures (fish housing and behavioral tests).

#### Y-Maze Test

**Figure 5:**
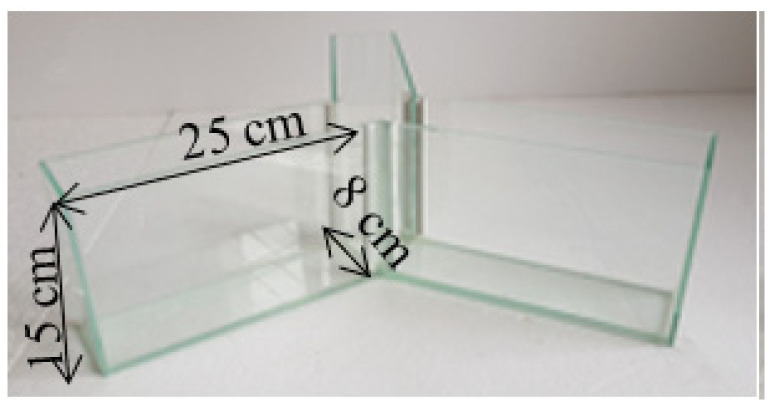
Y-maze glass tank.

Spatial memory and the response to novelty in zebrafish was assessed using the Y-maze task [36]. The location in the Y-maze task was considered to be a memory index [37]. The apparatus (Figure 5) consisted of a Y-maze glass tank with three identical arms (25 cm long, 8 cm wide, and 15 cm high), filled with 3 L of the home aquarium water. The water height in the Y-maze was 13 cm. Explicit geometric shapes (squares, circles, and triangles) were placed on the outer walls and were visible from the inside. The Y-maze test consisted of two trials separated by a 1 h interval. During the first trial, the fish could freely swim in the start arm and the other arm for 5 min while the novel arm was blocked by a dividing wall. In the second trial, the wall was removed, and the fish could explore for 5 min all three arms including the novel environment constituted by the novel arm. Fish were placed in different arms as starting points and the maze was rotated in each experiment to randomize the maze cues. The water was changed between groups and trials. Assessing time spent in each arm (percent of the total time), total distance traveled (m), and turn angle (°) were determined.

#### Novel Tank Diving Test (NTT)

**Figure 6:**
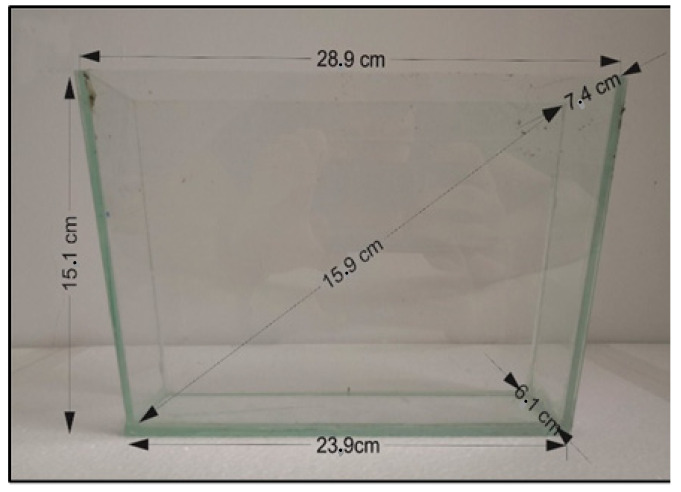
The NTT trapezoidal shaped glass.

In the NTT, the zebrafish exhibits robust behavioral responses to novelty-provoked anxiety. The NTT protocol applied in this study was described before by [38,39]. The testing apparatus (Figure 6) consisted of a trapezoidal shaped glass tank filled with 1.5 L of home tank water and having the following dimensions: 23.9 cm along the bottom × 28.9 cm at the top × 15.1 cm high with 15.9 cm along the diagonal side, 7.4 cm wide at the top and 6.1 cm wide at the bottom. The fish were individually placed in the testing tank and their behavior was recorded for 6 min with a webcam placed at 40 cm in the front of the tank. The tank was virtually divided into the top zone and bottom zone, respectively. To measure anxiety-like behavior and the locomotor activity of the zebrafish, we used the behavioral endpoints described previously by Cachat et al. [38].

### 4.8. Statistical Analyses

All results are expressed as mean ± standard error of the mean (S.E.M) and were analyzed by GraphPad Prism 9.0 software (GraphPad Software, Inc., San Diego, CA, USA). The normality of data distribution was evaluated using Shapiro-Wilk-Test. Datasets with multiple comparisons were evaluated using one-way or two-way ANOVA followed by Tukey’s post hoc test. *p* < 0.05 was considered to indicate a statistically significant difference.

## 5. Conclusions

All tested phenolic fractions and pure compounds (YuB and GloA) derived from the bark of *Yucca schidigera* showed significant in vivo locomotor improvement, memory enhancement and anxiolytic effects on the Sco-induced amnesia and anxiety level zebrafish models. The detailed phytochemical analysis of YS unpur, YS pur and YS poly revealed their chemical similarity, which is characterized by the presence of single or mixtures of spiro-flavostilbenoids. The promising neuroprotective effects of yucca phenolic preparations require deeper investigation.

## Figures and Tables

**Table 1:** Individual and total phenolic content (mg/g) of YS unpurified and purified phenolic fractions, using UHPLC-UV-MS.

N^o^	t_R_ (Min)	Compound	Formula	MW ^1^	YS Unpur ^2^	YS Pur ^3^
1	4.10	THMS ^4^	C_15_H_14_O_5_	274	48.85 ± 0.64	73.64 ± 1.14 ^5^
2	5.20	Dihydrokaempferol	C_15_H_12_O_6_	288	2.90 ± 0.37	3.52 ± 0.19
3	6.20	*Trans*-resveratrol	C_14_H_12_O_3_	228	8.12 ± 0.08	12.69 ± 0.20
4	9.63	Yuccaol E	C_30_H_22_O_10_	542	19.96 ± 0.31	31.06 ± 0.44
5	10.16	Yuccaol C	C_30_H_22_O_10_	542	66.34 ± 1.00	103.96 ± 1.46
6	10.45	Naringenin	C_15_H_12_O_5_	272	14.17 ± 1.28	22.30 ± 0.28
7	10.95	Yuccalide A	C_30_H_22_O_10_	542	11.51 ± 0.12	18.11 ± 0.49
8	11.05	Yuccaol D	C_30_H_22_O_10_	542	58.12 ± 0.83	90.42 ± 1.35
9	11.25	Kaempferol	C_15_H_10_O_6_	286	10.06 ± 0.41	14.34 ± 0.06
10	12.75	Yuccaol A	C_29_H_20_O_8_	496	14.63 ± 0.31	23.46 ± 0.31
11	12.94	Yuccaol B	C_29_H_20_O_8_	496	18.91 ± 0.37	29.83 ± 0.67
12	13.38	Gloriosaol E	C_45_H_30_O_15_	810	11.83 ± 0.21	18.79 ± 0.21
13	13.52	Gloriosaol D	C_45_H_30_O_15_	810	11.83 ± 0.21	18.78 ± 0.23
14	14.51	Gloriosaol A	C_45_H_30_O_15_	810	13.60 ± 0.19	21.53 ± 0.27
15	15.79	Gloriosaol C	C_45_H_30_O_15_	810	13.52 ± 0.46	21.00 ± 0.27
		Total amount			**324.34 ± 4.60**	**503.44 ± 7.46**

^1^ MW—molecular weight; ^2^ YS unpur—*Y. schidigera* unpurified phenolic fraction; ^3^ YS pur—*Y. schidigera* purified phenolic fraction; ^4^ THMS—*trans*-3,3′,5,5′-tetrahydroxy-4′-methoxystilbene; ^5^ Mean ± SD, *n* = 3.

## Data Availability

Data is contained within the article or Supplementary Materials.

## Supplementary Materials

The following supporting information can be downloaded at: https://www.mdpi.com/article/10.3390/molecules27123692/s1, Figure S1: Induced locomotor pattern and behavior in the Y-maze test of the *Yucca schidigera* substances in 3 doses level in scopolamine (Sco)-treated zebrafish. Representative locomotion tracking patterns of the control—untreated group; Sco (100 μM) treated group; groups treated with Sco (100 µM) and yucca (YS) preparations at 1, 3, 5 µg/L concentration levels: (a) YS pur—purified fraction; (b) YS poly (=YS pro)—polymeric fraction; (d) YuB—Yuccaol B; and (e) GloA—Gloriosaol A. Table S1: Acetylcholinesterase inhibition capacity (%) and EC_50_ (µg/mL) of the *Yucca schidigera* bark phenolic fractions. Table S2: Butyrylcholinesterase inhibition capacity (%) and EC_50_ (µg/mL) of the *Yucca schidigera* bark phenolic fractions. Table S3: 15-Lipoxygenase inhibition activity (%) and EC_50_ (µg/mL) of the *Yucca schidigera* bark phenolic fractions, yuccaol B, and gloriosaol A. Table S4: Cholinesterase inhibition (%) and IC_50_ values of the *Yucca schidigera* phenolic pure metabolites.

## Sample Availability

Samples of the plant materials, *Y. schidigera* unpurified, purified and polymeric fractions and the pure compounds yuccaols A-E, yuccalide A, gloriosaols A, C-E, *trans*-resveratrol, *trans*-3,3′,5,5′-tetrahydroxy-4′-methoxystilbene, dihydrokaempferol, naringenin, kaempferol are available from the authors Łukasz Pecio, Solomiia Kozachok and Wiesław Oleszek.
