# Supplementary material for: Neuroprotective Effect of Yucca schidigera Roezl ex Ortgies Bark Phenolic Fractions, Yuccaol B and Gloriosaol A on Scopolamine-Induced Memory Deficits in Zebrafish
Source: Molecules. 2022 Jun 8;27(12):3692. doi: 10.3390/molecules27123692 (PMC9227830; doi:10.3390/molecules27123692)
Supplement: Supplementary file 1 [file molecules-27-03692-s001.zip › molecules-1719254-supplementary.pdf]

molecules

Supplementary Materials

Neuroprotective Effect of *Yucca schidigera* Roezl ex Ortgies Bark Phenolic Fractions, Yuccaol B and Gloriosaol A on Scopolamine-Induced Memory Deficits in Zebrafish

Łukasz Pecio [1,2], Solomiia Kozachok [1,*], Ion Brinza [3], Razvan Stefan Boiangiu [3], Lucian Hritcu [3,*], Cornelia Mircea [4], Ana Flavia Burlec [5], Oana Cioanca [6], Monica Hancianu [6], Olga Wronikowska-Denysiuk [7], Krystyna Skalicka-Woźniak [2] and Wiesław Oleszek [1]

1 Department of Biochemistry and Crop Quality, Institute of Soil Science and Plant Cultivation—State Research Institute, Czartoryskich 8, 24-100 Puławy, Poland; lpecio@iung.pulawy.pl (Ł.P.); wieslaw.oleszek@iung.pulawy.pl (W.O.)
2 Department of Natural Products Chemistry, Medical University of Lublin, 20-093 Lublin, Poland; kskalicka@pharmacognosy.org
3 Department of Biology, Faculty of Biology, Alexandru Ioan Cuza University of Iasi, 700506 Iasi, Romania; ion.brinza@student.uaic.ro (I.B.); razvan.boiangiu@student.uaic.ro (R.S.B.)
4 Department of Pharmaceutical Biochemistry and Clinical Laboratory, Faculty of Pharmacy, "Grigore T. Popa" University of Medicine and Pharmacy, 16 University Street, 700115 Iasi, Romania; corneliamircea@yahoo.com
5 Department of Drug Analysis, Faculty of Pharmacy, "Grigore T. Popa" University of Medicine and Pharmacy, 16 University Street, 700115 Iasi, Romania; ana-flavia.l.burlec@umfiasi.ro
6 Department of Pharmacognosy, Faculty of Pharmacy, "Grigore T. Popa" University of Medicine and Pharmacy, 16 University Street, 700115 Iasi, Romania; oana.cioanca@gmail.com (O.C.); mhancianu@yahoo.com (M.H.)
7 Independent Laboratory of Behavioral Studies, Medical University of Lublin, 20-059 Lublin, Poland; olga.wronikowska-denysiuk@umlub.pl
* Correspondence: skozachok@iung.pulawy.pl (S.K.); hritcu@uaic.ro (L.H.); Tel.: +48-814 786- 882 (S.K.)

Citation: Pecio, Ł.; Kozachok, S.; Brinza, I.; Boiangiu, R.S.; Hritcu, L.; Mircea, C.; Burlec, A.F.; Cioanca, O.; Hancianu, M.; Wronikowska-Denysiuk, O.; et al. Neuroprotective Effect of *Yucca schidigera* Roezl ex Ortgies Bark Phenolic Fractions, Yuccaol B and Gloriosaol A on Scopolamine-Induced Memory Deficits in Zebrafish. *Molecules* **2022**, *27*, 3692. https://doi.org/10.3390/molecules27123692

Academic Editors: Tibor Kurtán and Jérôme Leprince

Received: 24 April 2022
Accepted: 6 June 2022
Published: 8 June 2022

Publisher's Note: MDPI stays neutral with regard to jurisdictional claims in published maps and institutional affiliations.

Copyright: © 2022 by the authors. Licensee MDPI, Basel, Switzerland. This article is an open access article distributed under the terms and conditions of the Creative Commons Attribution (CC BY) license (https://creativecommons.org/licenses/by/4.0/).

Abstract: *Y. schidigera* contains a number of unusual polyphenols, derivatives of resveratrol and naringenin, called spiro-flavostilbenoids, which have potent in vitro anti-inflammatory, antioxidant, and moderate cholinesterase inhibitory activities. To date, these compounds have not been tested in vivo for the treatment of neurodegenerative diseases. The aim of the present study was to evaluate the effects of both single spiro-flavostilbenoids (yuccaol B and gloriosaol A) and phenolic fractions derived from *Y. schidigera* bark on scopolamine-induced anxiety and memory process deterioration using a *Danio rerio* model. Detailed phytochemical analysis of the studied fractions was carried out using different chromatographic techniques and Nuclear Magnetic Resonance (NMR). The novel tank diving test was used as a method to measure zebrafish anxiety, whereas spatial working memory function was assessed in Y-maze. In addition, acetylcholinesterase/butyrylcholinesterase (AChE/BChE) and 15-lipooxygenase (15-LOX) inhibition tests were performed in vitro. All pure compounds and fractions under study exerted anxiolytic and procognitive action. Moreover, strong anti-oxidant capacity was observed, whereas weak inhibition towards cholinesterases was found. Thus, we may conclude that the observed behavioral effects are complex and result rather from inhibition of oxidative stress processes and influence on cholinergic muscarinic receptors (both 15-LOX and scopolamine assays) than effects on cholinesterases. *Y. schidigera* is a source of substances with desirable properties in the prevention and treatment of neurodegenerative diseases.

Keywords: *Yucca schidigera*; Asparagaceae; spiro-flavostilbenoids; stilbenoids; polyphenols; zebrafish; anxiety; memory; cholinergic function; oxidative stress

List of Supplementary Materials

Figure S1. Induced locomotor pattern and behavior in the Y-maze test of the *Yucca schidigera* substances in 3 doses level in scopolamine (Sco)-treated zebrafish. Representative locomotion tracking patterns of the control – untreated group; Sco (100 μM) treated group; groups treated with Sco (100 μM) and yucca (YS) preparations at 1, 3, 5 μg/L concentration levels: (**a**) YS pur – purified fraction; (**b**) YS poly (=YS pro) – polymeric fraction; (**d**) YuB – Yuccaol B; and (**e**) GloA – Gloriosaol A.

Table S1. Acetylcholinesterase inhibition capacity (%) and EC_{50} (μg/mL) of the *Yucca schidigera* bark phenolic fractions.

Table S2. Butyrylcholinesterase inhibition capacity (%) and EC_{50} (μg/mL) of the *Yucca schidigera* bark phenolic fractions.

Table S3. 15-Lipoxygenase inhibition activity (%) and EC_{50} (μg/mL) of the *Yucca schidigera* bark phenolic fractions, yuccaol B, and gloriosaol A.

Table S4. Cholinesterase inhibition (%) and IC_{50} values of the *Yucca schidigera* phenolic pure metabolites.

Figure S1. Induced locomotor pattern and behavior in the Y-maze test of the *Yucca schidigera* substances in 3 doses level in scopolamine (Sco)-treated zebrafish. Representative locomotion tracking patterns of the control – untreated group; Sco (100 µM) treated group; groups treated with Sco (100 µM) and yucca (YS) preparations at 1, 3, 5 µg/L concentration levels: (**a**) YS pur – purified fraction; (**b**) YS poly (=YS pro) – polymeric fraction; (**d**) YuB – Yuccaol B; and (**e**) GloA – Gloriosaol A.

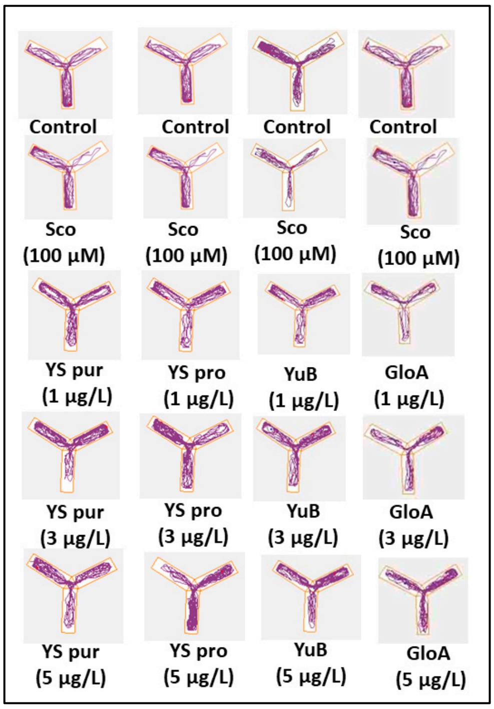

(a) **(b)** **(c)** **(d)**

Table S1. Acetylcholinesterase inhibition capacity (%) and EC_{50} (µg/mL) of the *Yucca schidigera* bark phenolic fractions.

**Sample**	**Concentration of sample (mg/mL)**								**EC_{50}, µg/mL**
	0.3125	*0.625*	*1.25*	*2.5*	*5*	*10*	*20*		
YS unpur	5.39 ± 0.07	8.29 ± 0.09	20.55 ± 0.1	38.66 ± 0.08	57.73 ± 0.09	92.14 ± 0.11	96.75 ± 0.08		188.76 ± 0.59
	0.0625	*0.125*	*0.25*	*0.5*	*1*	*2*	*4*	*8*	
YS pur	13.97 ± 0.04	19.80 ± 0.03	21.69 ± 0.07	23.36 ± 0.17	27.99 ± 0.04	32.62 ± 0.17	36.91 ± 0.15	46.17 ± 0.17	419.45 ± 0.86[a]
YS poly	12.09 ± 0.04	17.23 ± 0.05	19.91 ± 0.06	28.37 ± 0.08	34.64 ± 0.09	55.89 ± 0.07	74.64 ± 008	89.09 ± 0.14	82.51 ± 0.20[a]
Gal	16.25 ± 0.08	23.05 ± 0.16	30.58 ± 0.20	50.35 ± 0.15	69.23 ± 0.18	79.95 ± 0.10	92.27 ± 0.12	98.02 ± 0.17	24.69 ± 0.09

Notes: [a]sample vs galantamine p<0.0001 (statistically significant). YS unpur – *Y. schidigera* unpurified phenolic fraction; YS pur – *Y. schidigera* purified phenolic fraction; YS poly – *Y. schidigera* polymeric fraction; Gal – galantamine.

Table S2. Butyrylcholinesterase inhibition capacity (%) and EC_{50} (µg/mL) of the *Yucca schidigera* bark phenolic fractions.

**Sample**	**Concentration of sample (mg/mL)**								**EC_{50}, µg/mL**
	0.3125	*0.625*	*1.25*	*2.5*	*5*	*10*	*20*		
YS unpur	12.30 ± 0.15	20.26 ± 0.23	27.30 ± 0.18	41.80 ± 0.21	58.37 ± 0.48	88.81 ± 0.92	93.80 ± 0.25		176.14 ± 1.14
	0.0625	*0.125*	*0.25*	*0.5*	*1*	*2*	*4*	*8*	
YS pur	2.93 ± 0.11	4.88 ± 0.04	7.25 ± 0.11	14.05 ± 0.08	24.69 ± 0.12	34.16 ± 0.09	55.84 ± 0.08	78.77 ± 0.13	165.93 ± 0.44[a]
YS poly	7.28 ± 0.08	15.45 ± 0.10	20.25 ± 0.11	27.46 ± 0.08	36.77 ± 0.10	49.15 ± 0.11	62.63 ± 0.12	90.75 ± 0.18	104.46 ± 0.59[a]
Gal	15.05 ± 0.09	21.85 ± 0.10	31.09 ± 0.11	52.47 ± 0.18	70.69 ± 0.20	81.01 ± 0.17	92.45 ± 0.13	98.29 ± 0.17	23.07 ± 0.13

Notes: [a]sample vs galantamine p<0.0001 (statistically extremely significant). YS unpur – *Y. schidigera* unpurified phenolic fraction; YS pur – *Y. schidigera* purified phenolic fraction; YS poly – *Y. schidigera* polymeric fraction; Gal – galantamine.

Table S3. 15-Lipoxygenase inhibition activity (%) and EC_{50} (µg/mL) of the *Yucca schidigera* bark phenolic fractions, yuccaol B, and gloriosaol A.

**Sample**	**Concentration of sample (mg/mL)**								**EC_{50}, µg/mL**
	0.3125	*0.625*	*1.25*	*2.5*	*5*	*10*			
YS unpur	24.34±0.41	33.39±0.33	55.21±0.19	71.16±0.24	92.08±0.51	100±0			17.65±0.13
	0.0625	*0.125*	*0.25*	*0.5*	*1*	*2*	*4*	*8*	
YS pur	9.13 ± 0.33	12.40 ± 0.94	23.08 ± 0.77	48.67 ± 0.61	67.05 ± 1.26	98.93 ± 1.05	100 ± 0	100 ± 0	8.76 ± 0.21[a]
YS poly	9.67 ± 0.46	16.97 ± 0.38	25.55 ± 1.14	35.56 ± 0.80	64.42 ± 0.56	96.40 ± 1.09	100 ± 0	100 ± 0	12.78 ± 1.74[b]
YuB	12.95 ± 0.28	20.2 ± 0.86	29.84 ± 0.94	42.96 ± 0.53	75.67 ± 0.89	98.81 ± 0.68	-*	-*	9.66 ± 0.12[a]
GloA	7.45 ± 0.28	13.14 ± 0.47	25.74 ± 0.88	38.17 ± 0.95	59.04 ± 1.37	76.68 ± 1.65	100 ± 0	100 ± 0	12.34 ± 0.48[a]
Vit. C	10.92 ± 0.80	15.71 ± 0.31	20.84 ± 1.05	30.19 ± 0.79	40.67 ± 1.82	65.89 ± 1.25	94.19 ± 0.98	100 ± 0	21.52 ± 0.95

Notes: *insufficient sample quantity, [a]sample *vs* vitamin C $p<0.0001$ (statistically extremely significant); [b]sample vs vitamin C $p<0.0016$ (statistically highly significant). YS unpur – *Yucca schidigera* unpurified phenolic fraction; YS pur – *Y. schidigera* purified phenolic fraction; YS poly – *Y. schidigera* polymeric fraction.

Table S4. Cholinesterase inhibition (%) and IC_{50} values of *Yucca schidigera* phenolic pure metabolites.

No.	Compound	AChEi [1] (Inhibition % ± S.D. [2]) 1000 µM [3]	BChEi [4] (Inhibition % ± S.D. [2]) 1000 µM [3]
1	THMS [5]	19.33 ± 3.03 %	25.21 ± 2.58 %
2	Dihydrokaempferol	5.09 ± 2.38 %	8.56 ± 2.83 %
3	Naringenin	6.89 ± 1.82 %	21.07 ± 2.44 %
4	Galantamine [6]	97.92 ± 0.01% (IC_{50} = 2.29 ± 0.33 µM)	91.52±1.63 % (IC_{50} = 124.03 ± 4.05 µM)

[1] AChEi – acetylcholinesterase inhibition; [2] S.D. – Standard deviation; [3] Final concentration; [4] BChE – butyrylcholinesterase inhibition; [5] THMS – *trans*-3,3′,5,5′-tetrahydroxy-4′-methoxystilbene; [6] reference substance.
